# Complementary mNGS and traditional testing for bloodstream infections

**DOI:** 10.1515/med-2026-1494

**Published:** 2026-07-07

**Authors:** Dongjuan Chen, Xuemei Li, Zhenhui Wang, Liping Huang, Liu Qin

**Affiliations:** Department of Laboratory Medicine, Maternal and Child Health Hospital of Hubei Province, Tongji Medical College, Huazhong University of Science and Technology, Wuhan, China; Xinfeng County Maternal and Child Health Hospital of Jiangxi Province, Xinfeng County, China

**Keywords:** metagenomic next-generation sequencing (mNGS), bloodstream infection, blood culture, pathogen diagnosis, integrated diagnosis, complementarity

## Abstract

Bloodstream infections (BSIs) require rapid and accurate etiological diagnosis to guide timely antimicrobial therapy. Conventional diagnostic approaches, particularly blood culture, remain indispensable for antimicrobial susceptibility testing; however, they are limited by prolonged turnaround time and reduced sensitivity, especially following prior antibiotic exposure. Metagenomic next-generation sequencing (mNGS) has emerged as a culture-independent and hypothesis-free diagnostic tool capable of detecting a broad spectrum of pathogens directly from clinical samples. This approach is particularly advantageous for identifying rare, fastidious, and polymicrobial infections, as well as infections in immunocompromised patients. However, its clinical application remains constrained by challenges in distinguishing infection from colonization, interpreting antimicrobial resistance signals, and variability in bioinformatics pipelines. Thus, in the era of integrated diagnosis, mNGS does not replace but powerfully complements traditional methods. Furthermore, we propose a dynamic evidence-weighted integrated diagnostic framework to guide real time clinical decision and improve the clinical applicability of mNGS in bloodstream infections.

## Introduction and research value positioning

### Diagnostic challenges and clinical needs of bloodstream infections

Bloodstream infections (BSI) and sepsis pose a significant clinical burden globally, associated with substantial morbidity and mortality [1], 2]. Early effective antimicrobial therapy has been proven to improve patient outcomes, but the limitations of traditional culture methods hinder rapid diagnosis [2]. The main challenges currently faced in diagnosis include: reduced sensitivity of culture after antibiotic use [3], 4], difficulty in detecting non-culturable pathogens, and challenges in identifying emerging pathogens [5]. These challenges are particularly prominent in immunocompromised patients, whose risk of mixed infections and infections with rare pathogens is significantly increased [6], 7]. There is an urgent clinical need for diagnostic methods capable of rapidly and comprehensively identifying pathogens to guide precise anti-infective therapy [1], 8].

### Development history and principle overview of mNGS technology

Metagenomic Next-Generation Sequencing (mNGS) is an emerging broadband diagnostic technology that analyzes the nucleic acid content in patient samples through high-throughput sequencing, enabling unbiased detection and characterization of microbial DNA and/or RNA [9], 10]. This technology originated in the research field and has gradually transitioned to clinical applications [11]. The core advantage of mNGS lies in its hypothesis-independent detection approach, capable of simultaneously identifying various pathogens such as bacteria, viruses, fungi, and parasites [9], 12]. Plasma cell-free DNA (cfDNA) sequencing, as an important branch of mNGS, provides a new approach for bloodstream infection diagnosis by analyzing pathogen DNA fragments in circulation [4], 10], 13]. With the optimization of bioinformatics analysis pipelines, mNGS has demonstrated the potential for rapid diagnosis from sample to report within 24–48 h [14], 15].

### Clinical status and limitations of traditional diagnostic methods

Blood culture, as the gold standard for bloodstream infection diagnosis, still holds irreplaceable value in antimicrobial susceptibility guidance [14]. However, its limitations are increasingly evident: long culture cycles (typically requiring 2–5 days) [16], significantly reduced sensitivity after antibiotic use (from about 60 % to 30–40 %) [3], 4], and inability to detect non-culturable microorganisms [5]. Serological testing maintains a unique role in immune response assessment [14], but is limited by window period issues and cross-reactivity. For specific pathogens like Talaromyces marneffei, traditional culture methods are inefficient and time-consuming for diagnosis [17]. Multiple studies indicate that approximately 15–30 % of clinically relevant pathogens cannot be detected by existing conventional methods [5], 6], providing complementary space for new diagnostic technologies like mNGS. It is noteworthy that traditional methods still hold advantages in cost-effectiveness and standardization, especially in resource-limited areas [9], 18].

Despite the growing application of metagenomic next-generation sequencing (mNGS) in the diagnosis of bloodstream infections, a critical gap remains in translating its heterogeneous outputs into actionable clinical decisions. Emerging evidence suggests that integrating mNGS with conventional diagnostic methods within a structured, evidence-weighted framework may enhance the interpretability of diagnostic results and optimize clinical decision-making. Therefore, this review systematically summarizes the complementary roles of mNGS and traditional methods, and on this basis proposes a conceptual dynamic evidence-weighted integrated diagnostic framework (DEW-IDM) to inform the clinical management of bloodstream infections.

## Research progress in comparison of bloodstream infection diagnostic technologies

### Sensitivity/specificity comparison between mNGS and traditional culture methods

Multiple studies consistently demonstrate that mNGS shows higher sensitivity than blood culture, particularly in patients receiving prior antimicrobial therapy. Across published studies, the sensitivity of mNGS for bloodstream infection ranges from approximately 70 %–90 %, while specificity ranges from 75 % to 90 %, depending on study design and reference standards. For example, in a prospective cohort study including 209 patients with suspected bloodstream infection, using a composite clinical diagnosis as the reference standard, mNGS achieved a sensitivity of 87.1 % and specificity of 80.2 % [6]. In contrast, blood culture sensitivity in similar populations typically declines to 30–40 % after antibiotic exposure [19], 20]. Overall, current evidence suggests that mNGS provides improved detection sensitivity, whereas traditional culture remains essential for specificity confirmation and antimicrobial susceptibility testing.

### Methodological factors influencing mNGS performance

Current mNGS platforms can be broadly categorized into short-read and long-read sequencing technologies. Short read platforms offer high base accuracy and are widely used in clinical diagnostics [21], whereas long read platforms provide faster turnaround but are associated with higher error rates [22]. Host DNA depletion strategies significantly influence detection performance. Saponin based lysis preserves microbial DNA but may introduce bias, while nuclease based methods improve signal to noise ratio at the risk of pathogen DNA loss [23]. Sequencing depth also affects sensitivity and cost. Higher depth improves detection of low abundance pathogens but increases resource requirements [24]. Furthermore, bioinformatics pipelines including database selection and threshold settings play a critical role in determining diagnostic accuracy and false positive rates [19], 25].

### Detection efficiency differences among different sample types

Different sample types significantly impact the detection efficiency of mNGS. In patients with pulmonary infections, mNGS detection of bronchoalveolar lavage fluid (BALF) samples showed higher positivity rates and sensitivity, outperforming traditional microbiological detection methods [26]. Comparative studies on mNGS detection of cell-free DNA (cfDNA) and cellular DNA indicate that both detection strategies have their own advantages and need to be selected based on clinical circumstances [27]. In normally sterile body fluid (NSBF) testing, mNGS demonstrated faster detection speed and higher sensitivity compared to traditional culture methods [28]. It is noteworthy that the pathogen detection performance of mNGS in plasma samples varies depending on the pathogen type and infection site [29].

### Comparison of detection advantages for special pathogens

mNGS holds unique advantages in the detection of special pathogens. Research shows that viruses are the most common single or mixed infection type in CTD patients, particularly EB virus, CMV, and herpes simplex virus type 1 [10]. For fungal infections, mNGS can rapidly identify and distinguish fungal pathogens in positive blood culture specimens, which is crucial for timely initiation of appropriate antifungal therapy [29], 30]. In immunocompromised patients, the detection sensitivity of mNGS for bacteria and viruses is significantly better than traditional methods, reaching 100 % and 93.55 % respectively [26]. Furthermore, mNGS can effectively detect rare pathogens in bloodstream infections, which is of great significance for special pathogens difficult to detect by traditional culture methods.

## Unique value of mNGS in bloodstream infection management

### Rapid identification of rare/special pathogens

Studies indicate that mNGS has significant advantages in detecting pathogens that are difficult to culture or identify by traditional methods (Table 1), such as certain viruses, anaerobes, and special fungi [31]. Especially in acute leukemia patients, mNGS can effectively identify bloodstream infections caused by drug-resistant strains like Pseudomonas, providing critical diagnostic information for clinical practice [32]. This culture-independent detection method provides clinicians with a new tool for identifying rare pathogens [33].

**Table 1:** Comparative analysis of mNGS and conventional diagnostic methods.

Feature	mNGS	Conventional methods (blood culture)
Detection spectrum	Broad (bacteria, viruses, fungi, parasites)	Limited to culturable organisms
Sensitivity	High, especially after antibiotic exposure	Reduced after antibiotic use
Specificity	Moderate (affected by contamination and background DNA)	High
Turnaround time	24–48 h	2–5 days
Antimicrobial resistance	Genotypic prediction (ARGs)	Phenotypic AST (gold standard)
Mixed infection detection	Strong capability	Limited
Rare/fastidious pathogens	Detectable	Often missed
Quantitative interpretation	Relative abundance/reads-based	Colony count
Cost	High	Lower
Standardization	Limited, evolving	Well-established

### Comprehensive diagnostic capability for mixed infections

mNGS can not only detect single pathogens but also simultaneously identify multiple microorganisms present in a sample, revealing mixed infection situations. Compared to traditional targeted detection methods, mNGS can provide information on microbiome composition, helping to discover co-infections that may affect disease progression and prognosis [26]. In pediatric infection cases, mNGS detection of blood, central nervous system, and respiratory samples showed good positive and negative concordance rates, confirming its value in complex infection diagnosis [34]. Additionally, mNGS can detect pathogens that might be missed by traditional culture methods, such as certain fastidious bacteria and viruses [4], providing a new technical means for the comprehensive diagnosis of mixed infections.

### Simultaneous detection of antibiotic resistance genes

An important advantage of mNGS is its ability to simultaneously detect antibiotic resistance genes (ARGs), providing additional guidance information for clinical treatment [33]. Research shows that through optimized mNGS workflows, resistance genes associated with common antibiotic treatments can be successfully detected, such as those in Escherichia coli, Acinetobacter baumannii, and Group B Streptococcus [35]. This capability enables mNGS not only to identify pathogens but also to predict their potential resistance phenotypes. Currently, there are open-source cloud platform workflows (such as the CZ ID AMR module) specifically designed for integrating microbial and resistance gene detection from mNGS data, based on comprehensive antibiotic resistance databases and related software [36], 37]. It is noteworthy that resistance genes detected by mNGS need to be interpreted in conjunction with clinical manifestations, as some genes may originate from colonizing bacteria rather than true pathogens [38]. Despite this, the ability to simultaneously detect pathogens and resistance genes makes mNGS a highly promising diagnostic tool in bloodstream infection management [33], 35].

## Continued value and optimization directions of traditional methods

### The irreplaceability of culture methods in antimicrobial susceptibility guidance

Although culture methods have disadvantages such as being time-consuming (typically requiring 2–3 days) and labor-intensive [39], 40], their guiding value in antimicrobial susceptibility testing (AST) remains irreplaceable. Culture methods, through standardized techniques like broth microdilution, can provide accurate susceptibility results [41], which current molecular detection technologies cannot fully replace. Multiple studies indicate that culture methods, as the “gold standard” [2], 42], hold unique advantages in guiding clinical antibiotic selection and treatment optimization. Recent research has also focused on optimizing traditional culture processes, such as the EUCAST Rapid Antimicrobial Susceptibility Testing (RAST), which can shorten the detection time to within 8 h [43], 44], while maintaining accuracy comparable to traditional methods [45].

### The role of serological testing in immune response assessment

Bloodstream infections involve not only pathogen detection but are also closely related to the host’s immune response. Serological testing holds unique value in assessing systemic immune responses [46], providing information on host immune status that molecular detection cannot obtain. Especially in immunocompromised patients, serological markers can serve as important monitoring parameters for infection severity and treatment response. However, data on the specific application of serological testing in bloodstream infection management is relatively limited in the current literature, suggesting that this field requires more high-quality research to clarify its clinical value.

### Cost-effectiveness analysis and process optimization

The main challenges faced by traditional diagnostic methods include long detection cycles and limited sensitivity [47]. Multiple studies point out that for every 1-h delay in bloodstream infection diagnosis, patient mortality may increase by 7.6 % [48]. Therefore, optimizing the workflow of traditional methods has become a research focus, including: adopting rapid phenotypic detection technologies (such as the VITEK REVEAL system) [45], developing methods for direct detection from positive blood culture bottles [49], and improving sample collection and processing techniques [47]. Cost-effectiveness analysis shows that although molecular detection technologies are more expensive, optimized traditional methods (like EUCAST RAST) can significantly shorten detection times while maintaining lower costs [43], 44]. In resource-limited areas, optimized traditional methods remain a feasible choice [50].

## Construction and application of integrated diagnostic strategies

### Risk-stratified diagnostic approaches

Diagnostic strategies should be tailored according to patient risk profiles. In low-risk patients, a culture-first approach remains appropriate, whereas in high-risk or immunocompromised patients, early incorporation of mNGS may improve diagnostic yield [45], 51]. In critically ill patients, parallel testing strategies combining mNGS and conventional methods can minimize diagnostic delays.

### Dynamic evidence-weighted integration framework

**Figure 1:**
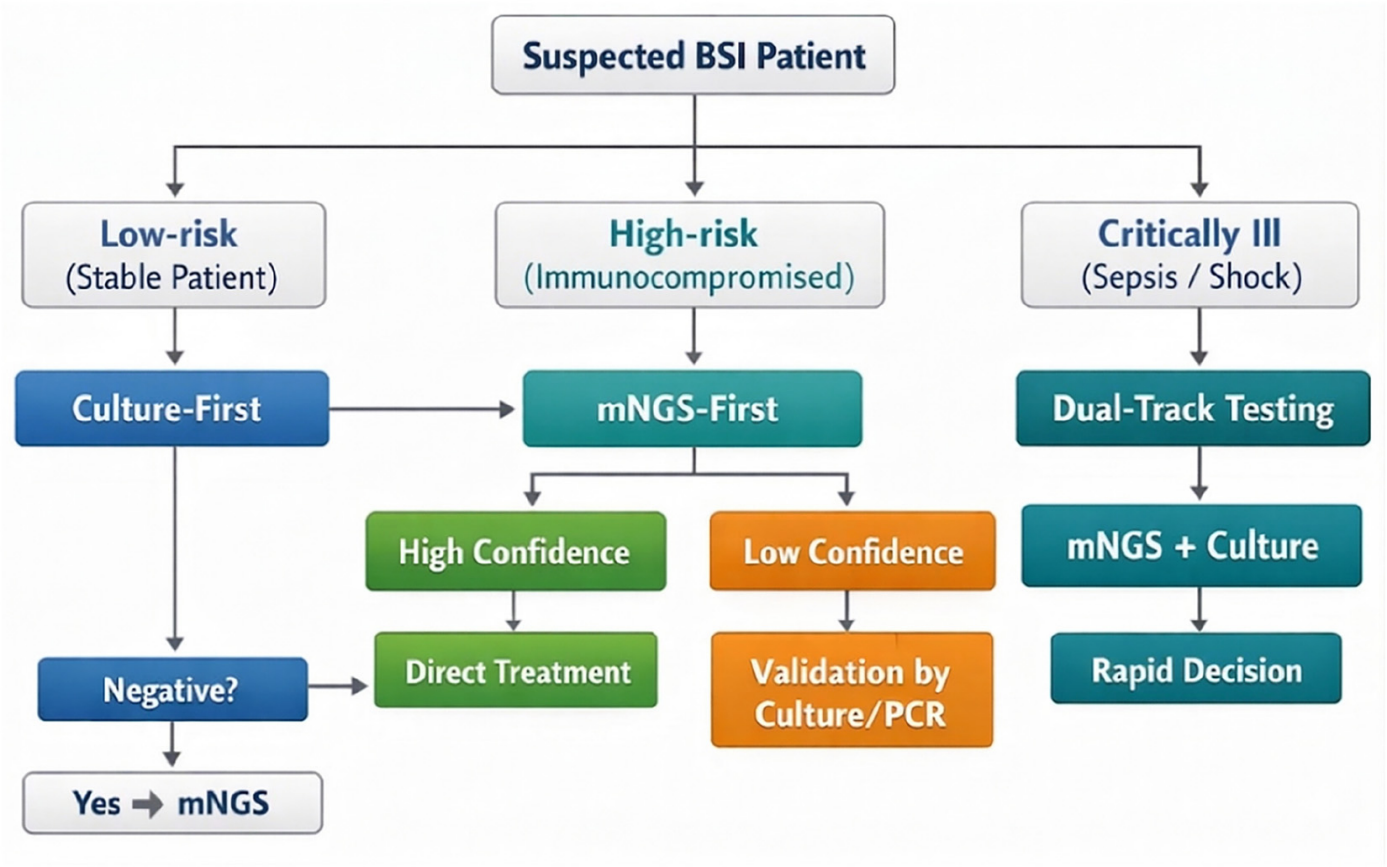
Dynamic evidence-weighted integrated diagnostic framework for bloodstream infections (DEW-IDM). This model integrates mNGS results with conventional microbiological diagnostics through multidimensional evidence weighting, incorporating pathogen characteristics, host factors, and clinical severity to guide real-time clinical decision-making.

To overcome the limitations of static diagnostic paradigms, we propose a dynamic evidence-weighted integration framework that incorporates mNGS results, pathogen characteristics, host immune status, and clinical severity (Figure 1). Within this framework, diagnostic findings are stratified into high-, intermediate-, and low-confidence categories, each linked to a corresponding clinical action.

To enhance clinical applicability, the framework is operationalized through a semi-quantitative scoring system. The DEW-IDM model follows a two-step process: (i) individual evidence domains are scored using predefined criteria (Table 2), and (ii) the aggregated score is translated into confidence levels and clinical recommendations (Table 3). This structure enables the transformation of heterogeneous diagnostic evidence into a reproducible and decision-oriented model for bloodstream infection (BSI) management.

**Table 2:** Semi-quantitative scoring system for the dynamic evidence-weighted integrated diagnostic model (DEW-IDM) in bloodstream infections.

Evidence domain	Parameter	Operational definition	Score	Evidence basis
mNGS quantitative signal	Pathogen-specific read count (RPM)	≥100 RPM	+2	Higher read counts correlate with true infection and microbial burden [52], [53], [54]
		10–99 RPM	+1	Intermediate signals require contextual interpretation [53], 55]
		<10 RPM	0	Low reads often represent background noise or contamination [54], 56]
Relative abundance	Dominance in microbial profile	≥30 %	+2	Dominant taxa more likely pathogenic rather than commensal [57], 58]
		5–29 %	+1	Possible pathogen; requires clinical correlation [57]
		<5 %	0	Likely background microbiota [58]
Clinical concordance	Consistency with syndrome	Fully consistent	+2	Clinical correlation essential for interpreting mNGS results [59], 60]
		Partially consistent	+1	May represent co-infection or atypical presentation [60]
		Inconsistent	0	Suggests colonization or false-positive signal [59]
Host status	Immunocompromised	Present	+1	Higher risk of opportunistic/mixed infections [61], 62]
		Absent	0	Lower pretest probability
Conventional microbiology	Blood culture concordance	Same pathogen identified	+3	Gold standard with highest specificity [63], 64]
		Negative/discordant	0	No confirmatory evidence
Contamination risk	Known contaminant taxa	Present	−2	Reagent/environment contamination is major false-positive source [56], 65]
		Absent	0	–
Antimicrobial resistance evidence	ARG detection concordant with phenotype	Present	+1	mNGS enables ARG detection but requires cautious interpretation [66], 67]
		Absent/uncertain	0	Limited reliability

**Table 3:** Interpretation of total scores and corresponding clinical recommendations in the DEW-IDM model.

Total score	Interpretation	Clinical recommendation
≥6	High-confidence infection	Initiate or adjust targeted antimicrobial therapy
3–5	Intermediate-confidence	Perform targeted validation (culture, PCR) and reassess clinically
≤2	Low-confidence (colonization/contamination likely)	Avoid overtreatment; monitor and correlate clinically

The scoring system integrates multidimensional evidence across four principal domains: (i) mNGS-derived quantitative metrics, including pathogen-specific read counts (reads per million, RPM) and relative abundance; (ii) clinical concordance between detected pathogens and patient presentation; (iii) host-related factors, particularly immunocompromised status; and (iv) conventional microbiological validation, primarily blood culture results. In addition, contamination risk and antimicrobial resistance gene (ARG) detection are incorporated as modifying factors to refine interpretation.

The weighting of these domains reflects their relative diagnostic value. Quantitative sequencing metrics and microbial dominance are prioritized, as higher pathogen burden is consistently associated with increased likelihood of true infection [52], [53], [54]. Clinical concordance is explicitly incorporated to contextualize sequencing findings, addressing the intrinsic limitation of mNGS in distinguishing infection from colonization based solely on nucleic acid detection [59], 60]. Host immune status serves as a contextual modifier, given the higher prevalence of opportunistic and polymicrobial infections in immunocompromised patients [61], 62].

Concordant blood culture results are assigned the highest weight, reflecting their role as the reference standard for pathogen confirmation and antimicrobial susceptibility testing [63], 64]. In contrast, taxa commonly associated with environmental or reagent contamination are negatively weighted to mitigate false-positive interpretations, particularly in low-biomass samples [56], 65]. ARG detection is incorporated as supportive evidence with modest weighting, acknowledging the known discordance between genotypic resistance markers and phenotypic susceptibility [66], 67].

Based on cumulative scores, findings are stratified into three categories. High-confidence results support immediate initiation or adjustment of targeted antimicrobial therapy. Intermediate-confidence findings warrant targeted validation using conventional methods such as culture or polymerase chain reaction. Low-confidence signals are interpreted cautiously, emphasizing clinical observation and avoidance of unnecessary antimicrobial escalation.

This structured approach directly addresses a central challenge in mNGS-based diagnostics – the differentiation of true infection from colonization or contamination – by integrating quantitative sequencing data with clinical and microbiological context. The DEW-IDM framework provides a transparent and adaptable basis for clinical decision-making and offers a practical foundation for future prospective validation.

## Current challenges and future directions

### Standardization and source-stratified false positives

One of the major barriers to the clinical implementation of mNGS is the lack of standardization across the entire diagnostic workflow, spanning sample processing, sequencing strategies, and bioinformatics analysis. These sources of variability not only affect analytical performance but also directly contribute to heterogeneous false-positive signals, which represent a central challenge in clinical interpretation. Importantly, false positives in mNGS should not be regarded as a uniform phenomenon; rather, they arise from distinct biological and technical origins, including environmental contamination, commensal colonization, translocation of microbial cell-free DNA, and bioinformatics misclassification. A source-stratified understanding of these signals is therefore essential for developing clinically actionable interpretation frameworks. This challenge is most prominently reflected in the difficulty of distinguishing colonization from true infection, which has been consistently identified as a major limitation of mNGS in clinical studies. Because mNGS indiscriminately captures all nucleic acids present in a sample, detection of a microorganism does not necessarily indicate pathogenicity. In bloodstream and sterile-site diagnostics, this limitation may lead to overdiagnosis or inappropriate antimicrobial escalation if results are interpreted without sufficient clinical context. Recent efforts have attempted to mitigate this issue through quantitative and context-aware interpretation strategies. For instance, metagenomic studies have demonstrated that quantitative profiling of specific taxa can improve discrimination between colonization and infection, with microbial abundance metrics showing greater predictive value than conventional culture in certain settings [20], 68]. However, such approaches remain non-standardized, and clinically validated thresholds for distinguishing pathogenic from non-pathogenic signals are lacking. Despite these limitations, mNGS has demonstrated tangible clinical impact on therapeutic decision-making. In a cohort of 107 patients with suspected infection, mNGS identified pathogens not detected by conventional methods in approximately 30 % of cases, leading to antimicrobial regimen modifications in 28 % of patients, including both de-escalation and escalation strategies [69]. These findings highlight the potential of mNGS to refine antimicrobial therapy; however, they also underscore the reliance on clinician-dependent interpretation in the absence of standardized decision algorithms. The challenge of interpretation extends further to antimicrobial resistance gene (ARG) detection, where mNGS enables comprehensive resistome profiling but introduces additional layers of uncertainty. While mNGS can detect a wide range of ARGs and capture dynamic changes in resistance gene abundance following antibiotic exposure, the link between detected resistance genes and phenotypic resistance remains inconsistent. This discrepancy is particularly evident for low-abundance genes or ARGs originating from non-pathogenic or colonizing organisms. Advanced bioinformatics tools [70], [71], [72]. such as pipelines that associate ARGs with their putative microbial hosts, have been developed to improve interpretability, yet their clinical validation remains limited. Taken together, these challenges highlight that the primary bottleneck in mNGS is no longer detection sensitivity, but rather the lack of standardized, source-aware interpretative frameworks. Future efforts should focus on integrating quantitative microbial signals with host-response biomarkers, establishing unified thresholds for pathogen reporting, and developing validated bioinformatics pipelines capable of distinguishing contamination, colonization, and true infection. Such advances will be critical for transforming mNGS from a high-sensitivity exploratory tool into a reliable and clinically actionable diagnostic modality. Future prospective studies are warranted to validate and refine the proposed scoring thresholds and weighting coefficients in diverse clinical settings.

### Bioinformatics optimization and the role of artificial intelligence

Bioinformatics analysis represents a critical bottleneck in mNGS workflows. Current pipelines rely heavily on predefined thresholds and reference databases, which may not adequately account for clinical variability. Artificial intelligence (AI) offers promising solutions by enabling pattern recognition across large datasets and integrating sequencing results with clinical variables. AI-assisted models may improve pathogen prioritization, reduce false positives, and provide real-time clinical decision support.

### Future research directions: toward practical implementation

Future research should move beyond descriptive studies and focus on clinically actionable outcomes. Key priorities include: (1) Prospective interventional trials evaluating mNGS-guided therapy. (2) Development of resource-stratified diagnostic workflows. (3) Integration of multi-omics data for improved specificity. (4) Standardization of reporting criteria and interpretation guidelines. In addition, simplified mNGS-centered workflows may be explored in resource-limited settings, challenging the traditional reliance on culture-based infrastructure.

## Summary and core conclusions

mNGS represents a transformative advancement in the diagnosis of bloodstream infections, offering unparalleled breadth of pathogen detection. However, its clinical utility remains highly dependent on appropriate interpretation and integration with conventional diagnostic methods. Structured, evidence-based frameworks such as the proposed DEW-IDM model may help maximize the complementary strengths of both approaches and support more consistent clinical decision-making. Ultimately, the future of bloodstream infection diagnostics lies in the development of adaptive, integrated diagnostic systems that move beyond single-modality testing to improve patient outcomes.
